# Non-Visual Effects of Light on Melatonin, Alertness and Cognitive Performance: Can Blue-Enriched Light Keep Us Alert?

**DOI:** 10.1371/journal.pone.0016429

**Published:** 2011-01-26

**Authors:** Sarah Laxhmi Chellappa, Roland Steiner, Peter Blattner, Peter Oelhafen, Thomas Götz, Christian Cajochen

**Affiliations:** 1 Centre for Chronobiology, Psychiatric Hospital of the University of Basel, Basel, Switzerland; 2 The CAPES Foundation/Ministry of Education of Brazil, Brasilia, Brazil; 3 Department of Physics, University of Basel, Basel, Switzerland; 4 Federal Office of Metrology (METAS), Bern-Wabern, Switzerland; Ecole Polytechnique Federale de Lausanne, Switzerland

## Abstract

**Background:**

Light exposure can cascade numerous effects on the human circadian process via the non-imaging forming system, whose spectral relevance is highest in the short-wavelength range. Here we investigated if commercially available compact fluorescent lamps with different colour temperatures can impact on alertness and cognitive performance.

**Methods:**

Sixteen healthy young men were studied in a balanced cross-over design with light exposure of 3 different light settings (compact fluorescent lamps with light of 40 lux at 6500K and at 2500K and incandescent lamps of 40 lux at 3000K) during 2 h in the evening.

**Results:**

Exposure to light at 6500K induced greater melatonin suppression, together with enhanced subjective alertness, well-being and visual comfort. With respect to cognitive performance, light at 6500K led to significantly faster reaction times in tasks associated with sustained attention (Psychomotor Vigilance and GO/NOGO Task), but not in tasks associated with executive function (Paced Visual Serial Addition Task). This cognitive improvement was strongly related with attenuated salivary melatonin levels, particularly for the light condition at 6500K.

**Conclusions:**

Our findings suggest that the sensitivity of the human alerting and cognitive response to polychromatic light at levels as low as 40 lux, is blue-shifted relative to the three-cone visual photopic system. Thus, the selection of commercially available compact fluorescent lights with different colour temperatures significantly impacts on circadian physiology and cognitive performance at home and in the workplace.

## Introduction

The non-visual effects of ocular light at short wavelengths strongly impinge on the human circadian timing system [1], [2], most probably via novel photoreceptors with the photopigment melanopsin [3], [4], [5]. Maximal response of this non-image-forming (NIF) system to light occurs between 446 and 483 nm for melatonin suppression [6], [7]. Furthermore, circadian phase shifts seem to be more sensitive to 460-nm light compared to 555-nm light at high irradiances [8]. Repercussions on human physiology include increased heart rate and core body temperature after blue (460 nm) but not after green light (550 nm) of equal photon density when administered in the evening [9], together with decreased electroencephalographic (EEG) slow-wave activity in the first cycle of non- rapid eye (NREM) sleep and shortened rapid eye movement (REM) sleep duration in the first two cycles [10].

Neurobehavioral responses triggered by light exposure encompass improved alertness and performance [11], [12], as indexed by specific cortical responses to cognitive tasks in Photon Emission Tomography [13] and functional Magnetic Resonance Imaging (fMRI) techniques [12]. Recent fMRI studies have demonstrated that daytime exposure to blue light, when compared to green [14] or violet [15], is more effective in enhancing responses to a memory task in several cortical, thalamic and brainstem areas. Thus, besides phase shifting the human circadian clock scheduled exposure to light provides an effective tool for improving cognitive performance [16]. In all the above mentioned studies, volunteers were tested under stringently controlled conditions, which are crucial for determining spectral sensitivity, but leave open the question of whether this sensitivity could be utilized in more practical scenarios to shift circadian rhythms, to alleviate jet lag and shift work symptoms, and to improve alertness and cognitive performance at the workplace and at house settings [17].

Lamps and light-producing devices emitting exclusively or relatively more short-wavelength energy are now commercially available [18]. Compact fluorescent (CF) lamps that provide correlated lamp colour temperature (CCT) [in kelvin (K)], that indicate the relative proportion of warm versus cool colours in a light source, are very often sold, because of the low energy consumption and governmental regulations to replace traditional incandescent bulbs.

Considering the mounting evidence that non-rod and non-cone photoreceptors might form the basis of the non-image forming photoreceptive pathway mediating both the circadian and direct effects of light, we hypothesized that the acute effect of light on melatonin, alertness, and cognitive performance is blue-shifted, such that CF lamp with 6500K (“cold light”) induce greater melatonin suppression and an enhanced alerting effect than CF lamp with 2500K (“warm light”) and incandescent light lamp of 3000K (“classic light”). Our two main hypotheses were as follows:

1. During 2-hour exposure to light in the evening, CF lamp with light at 6500K will attenuate the expression endogenous melatonin levels, in comparison to light at 2500K and at 3000K;
2. CF lamp with light at 6500K will promote an augmentation of subjective and objective alertness levels when compared to light at 2500K and at 3000K during and after 2-hour exposure to light in the evening. Furthermore, it will have an overall effect of enhancing alertness and performance in cognitive tasks specifically associated with sustained attention.

## Methods

### Study participants

Study volunteers were recruited through advertisements at different Swiss universities. All potential participants were questioned about sleep quality, life habits and health state. Only candidates with a Pittsburgh sleep quality index (PSQI) score <5 [19] and without extreme chronotype ratings [>14 and <21 points on the morning-evening M/E questionnaire [20]] were enrolled in this study. Sixteen male volunteers (age range, 20–28 years; mean 24.3±2.1 SD) were enrolled in this study. All participants were non-smokers, free from medical, psychiatric, and sleep disorders as assessed by history, a medical and biochemistry examination. Furthermore, a comprehensive toxicological analysis of urine for prescription medication, non-prescription medication and drug abuse was carried out prior to the beginning of the study, along with an ophthalmologic examination in order to exclude volunteers with visual impairments. All participants were instructed to abstain from excessive alcohol and caffeine consumption (i.e., at most 5 alcoholic beverages per week, and 1 cup of coffee or 1 caffeine-containing beverage per day). Exclusion criteria also included shift work within the last 3 months, body mass index <19 and >28, and transmeridian flights during the month prior to study. Furthermore, they were instructed to keep a regular sleep-wake schedule (bedtimes and wake times within±30 min of self-selected target time). Compliance to this outpatient segment of the study was verified by wrist actigraphy (actiwatch L, Cambridge Neurotechnologies, Cambridge, UK) and self-reported sleep logs. All volunteers gave written informed consent. The protocol, advertisements, screening questionnaires, and consent form were approved by the local Ethical Committee (EKBB/Ethikkommission beider Basel, Switzerland) and were in agreement with the Declaration of Helsinki.

### Study protocol

**Figure 1:**
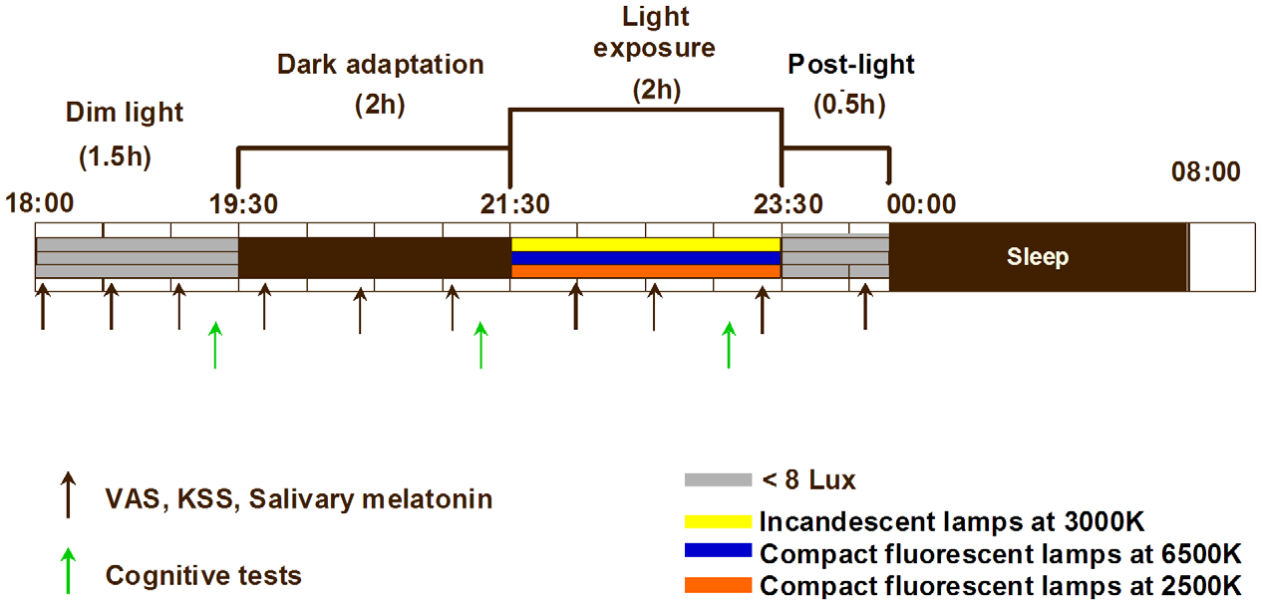
Protocol design. Light exposure at 6500K (blue bar), 2500K (orange bar) and 3000K (yellow bar).

The study consisted of three segments, performed in a balanced cross-over design, separated by a 1-week intervening period (Fig. 1). On the basis of the volunteers' habitual bedtimes, the protocol started 10 h after usual wake-up time in the early evening (i.e. 18:00 h) and ended the next day, after usual wake-up time (i.e. 08:00 h). Participants underwent an episode of 1.5 h under dim light conditions (<8 lux), which was followed by a 2-h dark adaptation episode under complete darkness (0 lux). Subsequently, light exposure was initiated for the next 2 h. During this 2-h episode, participants received light from either CF lamp with 6500K or 2500K or incandescent light bulb at 3000K. Afterwards, participants remained awake for approximately 1-h episode under dim light conditions (<8 lux), before a sleep episode of approximately 8 h. The treatment order (6500 vs. 2500K vs. 3000K) was counter-balanced in order to avoid possible order effects of the light treatment. The entire control protocol was conducted at the same time of day (evening), with the same light intensity, during all sessions and on all subjects.

### Lighting settings

**Figure 2:**
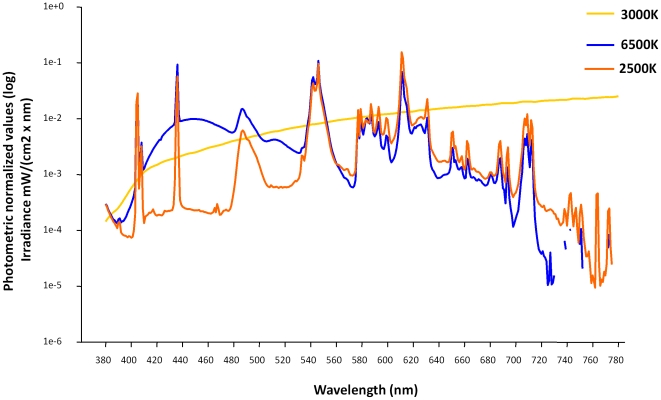
Spectral composition (light wavelength by Irradiance µV^2^/(cm^2^x nm) of light at 6500K (blue line), 2500K (orange line) and 3000K (yellow line).

A commercially available compact fluorescent light source with a highly correlated colour temperature (6500 K, Osram Duluxstar mini twist, Lumilux, cool daylight, France) was compared with a similar light source with lower colour temperature (2500 K, Osram Duluxstar mini twist, Lumilux, warm comfort light) and with an incandescent light source with a colour temperature of 3000 K (Osram incandescent lamp Classic A). Light was homogenously distributed within the entire field of view, since the room was very uniformly painted with high reflective white painting. Thus, the whole room acts almost like an integrating sphere. Both types of fluorescent lamps and the incandescent lamp were 18 W, with spectral range from 380–780 nm, and had a similar spectral power distribution at wavelengths above 530 nm (Fig. 2). However, the 6500 K light source produced a considerably higher output between 420 to 520 nm (although the peak at 435 nm was similar). The photon flux was not the same for all light sources, since the incandescent lamp emits more IR-photons, which are not relevant. The action spectra were normalized to the photometric values, which mean that the luminance was the same for all lights, such that all light conditions have the same brightness for the participants. Spectral composition was compared with the 6500 K, 2500K and 3000K, as shown in figure 2. Illuminance at the eye level was in the order of 38 to 40 lux. Mean illuminance levels measured at the wall were 40 lux and the wall reflectance was about 0.95 in the visible wavelength range. The average luminance of see by the test person within his field of view is therefore of 12 cd/m2. A calibrated high quality luminance meter (LMT L 1009, LMT Lichtmesstechnik GMBH Berlin) was utilized in order to ascertain that the light sources were adapted to have the same luminance. During the study protocol, subjects remained seated in a chair that was facing the wall (at a distance of 95 cm), and the light source was placed behind them, in order to minimize any possible visual constraints. During dim light exposure, dark adaptation, light exposure and post-light exposure they filled in questionnaires (described in the following sub-section) and the cognitive tasks (in sub-section “cognitive performance”).

### Subjective assessment of alertness, well-being and visual comfort

Subjective sleepiness was assessed every 40 minutes on the Karolinska Sleepiness Scale (KSS) [21] along with visual analogue scales (VAS) were for mood and alertness on a 100 mm scale. For the assessment of subjective well-being, since the direction of the extremes was not the same for all the 3 items, the formula utilized was as follows [22]: subjective well-being  =  [VAS mood + (100 –VAS tension) + VAS physical comfort]/3.

The Mental Effort Rating Scale (MERS) [23], which involves perception of mental strain, concentration and tiredness, was administrated approximately 20 minutes after each condition (dim light, dark adaptation, light exposure and post-light exposure) in order to access, respectively, the participants' visual comfort and their mental effort as a response to the lighting conditions. The Visual Comfort Scale (VCS) [24], which comprises a visual analogue scale on a 100 mm scale, comprises (1) visual well-being and comfort and (2) glare and brightness, was administered simultaneously with the MERS. In this questionnaire, glare and brightness are probed as, respectively, “Does the light have less glare or more?” and “Is the light too dark or bright?” More glare and brightness are conceived as helping to visualize patterns and/or to read, although high levels of glare and brightness can point to potentially less comfortable light perception in a given environmental light setting. The VAS, VCS and MERS were carried out prior to the cognitive tests.

### Salivary melatonin

Saliva collections were scheduled during wakefulness every 40 minutes. A direct double-antibody radioimmunoassay was utilized for the melatonin assay (validated by gas chromatography–mass spectroscopy with an analytical least detectable dose of 0.65 pm/mL; Bühlmann Laboratory, Schönenbuch, Switzerland) [25]. The minimum detectable dose of melatonin (analytical sensitivity) was determined to be 0.2 pg/ml.

### Cognitive performance

The psychomotor vigilance task and the GO/NOGO task, both of which are directly related to sustained attention, and the Paced Visual Serial Addition Task, which comprises a task of executive function, were administered during dim light, dark adaptation and light exposure.

The Psychomotor vigilance Task (PVT) is a sustained attention performance task known to be sensitive to sleep loss and circadian rhythmicity [26], [27]. The study participants were required to quickly press a button as soon as an auditory stimulus occurred, which were presented in intervals randomly varying from 3 to 7s. All participants were instructed to press the response button as fast as possible as soon they heard an auditory stimulus, and the duration of the PVT lasted 5 minutes.

The Paced Visual Serial Addition Task (PVSAT) [28] is an addition task heavily dependent on frontal brain regions, which involves higher order executive functioning. Single digits (1 to 9) appear on screen and each must be added to the digit which preceded it, and the resulting answer is selected from an on-screen numerical keypad (“sum” of adjacent pairs, not a total across all digits presented). Digits were seen for 1000 ms, with an interval of 2000 ms between them.

The GO/NOGO task [29] was used to measure the capacity for sustained attention and response control. In this test, participants had to press the space bar within 0.5 second if the letter “M” was shown on the screen, and they were reminded by an auditory stimulus to press faster if their response was above 0.5 seconds. If the letter “W” was shown, participants were instructed not to press any buttons. A total of 80% of “M” letters were shown in a random sequence. Approximately 200 “M”s were shown during 8 minutes.

The cognitive tasks (PVT, PVSAT and GO/NOGO) had stimulus presentations randomized afresh for each test use and were administered in the same order for each participant (PVT, PVSAT and GO/NOGO). In this study, sustained attention was indexed by the PVT and GO/NOGO performance, while executive functioning comprised performance on the PVSAT.

### Statistical Analysis

For all analysis, the statistical package SAS (SAS Institute Inc., Cary, NC, USA; Version 6.12) was utilized. Statistical analysis of the time course were carried out for each variable (subjective sleepiness, well-being, mental effort, visual comfort, PVT, GO/NOGO and salivary melatonin) with the mixed-model analyses of variance for repeated measures (PROC MIXED) with factors “light condition” (6500K vs. 2500K vs. 3000K) and “session” (dim light, dark adaptation and light exposure). Alpha adjustment for multiple comparisons was applied in PROC MIXED using Tukey-Kramer test. For the PVT performance, the default performance metrics - median reaction time (RT), 10% fastest and 10% slowest RT and lapses - and the interpercentile range between the 10th and 90th percentile were calculated according to [27]. Pearson correlations were utilized between salivary melatonin and PVT performance and the GO/NOGO task. All *p-*values derived from r-ANOVAs were based on Huynh-Feldt's (H-F) corrected degrees of freedom (significance level: *p*<0.05).

## Results

### Subjective sleepiness, well-being, mental effort and visual comfort

**Figure 3:**
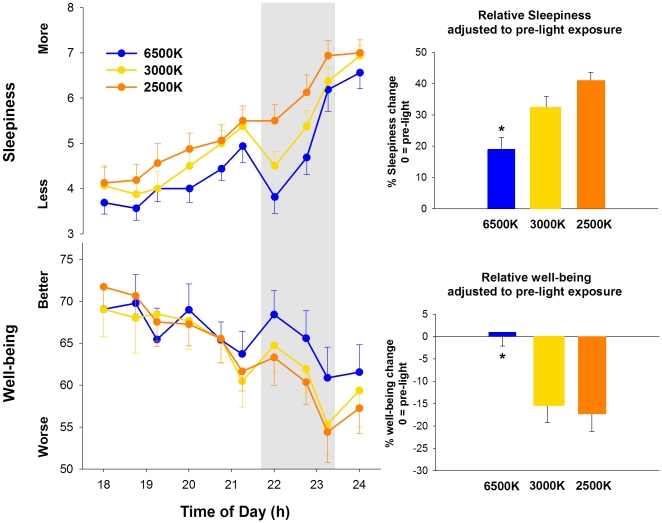
Sleepiness and well-being during pre-light, 2-h light (grey bar) at 6500K, 2500K and 3000K, and post-light. * p<0.05.

The time course of subjective sleepiness is illustrated in Fig. 3 (upper panel). The r-ANOVA yielded significant differences for the main factors “light condition” (F_2,24_ = 18.3, p<0.001) and “session” (F_9,35_ = 35.5, p<0.001). Comparison of subjective sleepiness across different light conditions indicated that light at 6500K, 3000K and 2500K resulted, respectively, in an increase of 19±3.8%, 32±4.1% and 41±2.6% in comparison to pre-light exposure (1-way ANOVA, F_2,15_ = 2.8, p = 0.04) (Fig. 3, upper inset).

For subjective well-being (Fig. 3, bottom panel), significant differences were elicited by the r-ANOVA for the main factors “light condition” (F_2,24_ = 17.1, p<0.05), “session” (F_9,35_ = 31.8, p<0.001) and the interaction of factors “light condition” and “session” (F_18,21_ = 0.6, p = 0.04). *Post-hoc* comparisons yielded a significant increase in subjective well-being during light exposure at 6500K in comparison to 2500K and 3000K, starting approximately 30 minutes after lights on. When comparing subjective well-being during light exposure to pre-light levels, light at 6500K, 3000K and 2500K resulted, respectively, in an increase of 1.1±3.1%, and a decrease of 15±4.2% and 17±4.1% in comparison to pre-light exposure (1-way ANOVA, F_2,12_ = 2.4, p = 0.03) (Fig. 3, lower inset).

**Figure 4:**
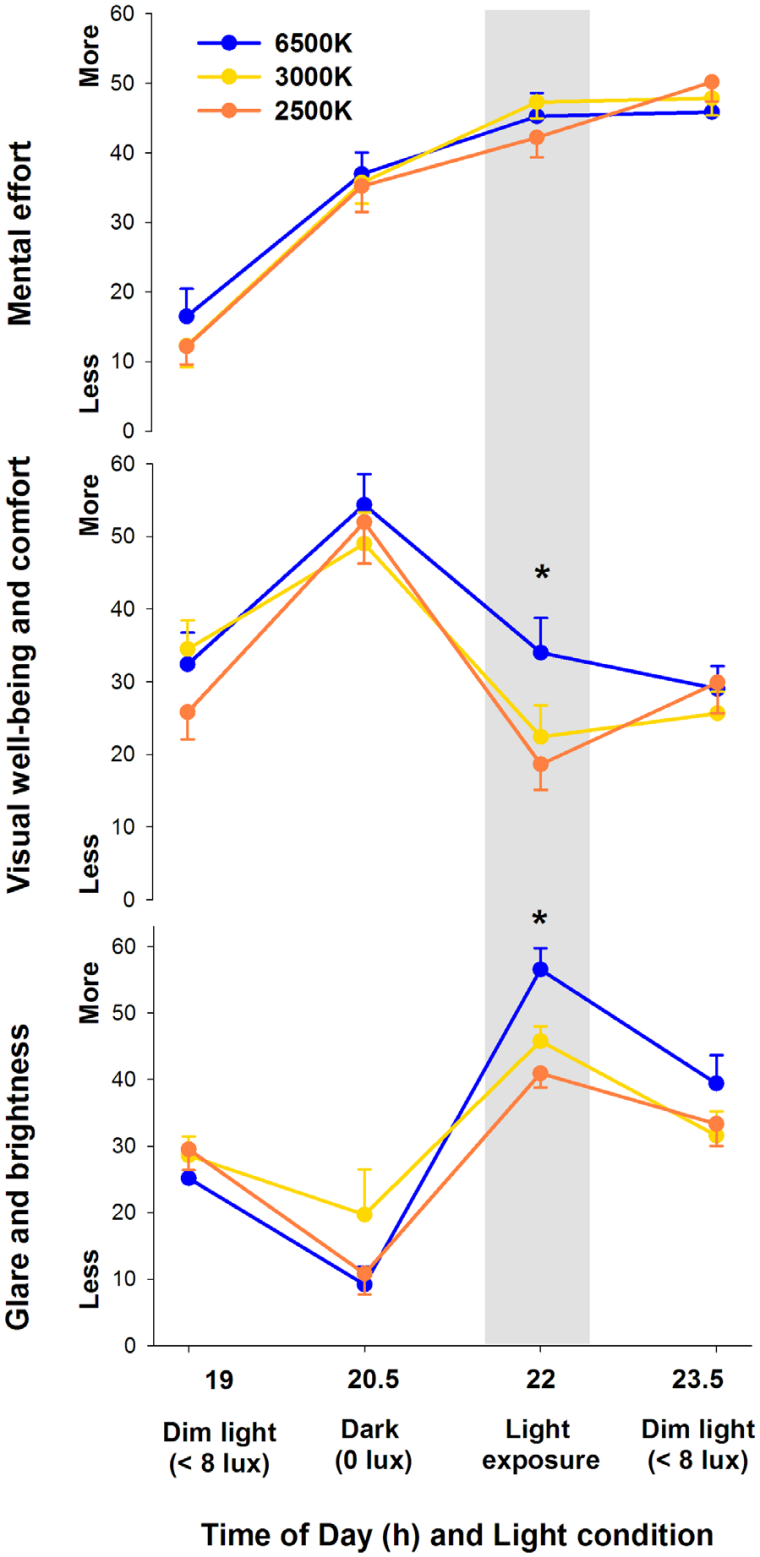
Mental effort, visual well-being and glare/brightness during pre-light, 2-h light (grey bar) at 6500K, 2500K and 3000K, and post-light. * p<0.05.

Mental effort did not significantly differ between sessions and light conditions (Fig. 4, upper panel). On the other hand, visual well-being and comfort (Fig. 4, middle panel) differed in a wavelength-dependent manner, as indicated by a significant two-way interaction of the factors “light condition” and “session” (F_6,17_ = 1.4, p = 0.04). *Post-hoc* comparisons yielded a significant increase in visual well-being and comfort during light at 6500K in comparison to 3000K and 2500K. Similarly, a significant two-way interaction of the factors “light condition” and “session” was observed for visual glare and brightness (F_6,17_ = 2.8, p = 0.01; Fig. 4, bottom panel), with a significant increase during light at 6500K in comparison to 3000K and 2500K.

### Cognitive performance

PVSAT performance (i.e. number of correct responses) did not yield differences for the main factors “light condition” and “session”, as well as for the interaction of these two factors. On the other hand, main factors “session” yielded a tendency for reaction time (RT; F_2,16_ = 2.7, p = 0.06), while “light condition” yielded significant differences for RT (F_2,24_ = 3.1, p = 0.04), with faster RT to light exposure at 6500K in comparison to 2500K and 3000K. However, the interaction of factors “light condition” and “session” did not reach significance.

**Figure 5:**
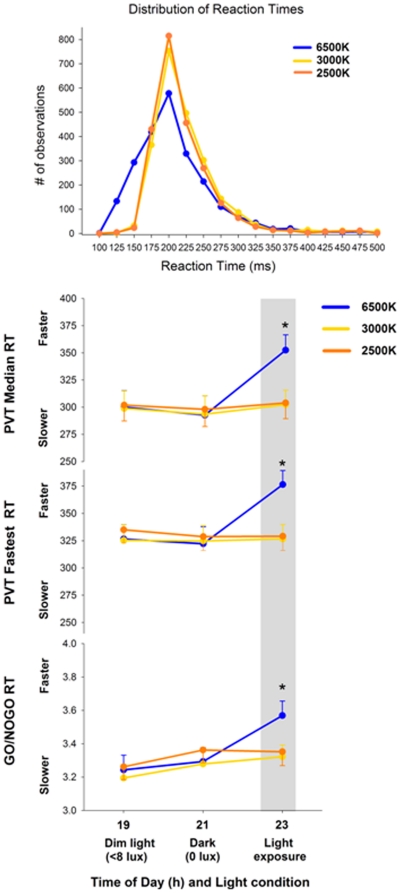
Distribution of RT, PVT median RT, PVT fastest RT and GO/NOGO RT during pre-light, 2-h light (grey bar) at 6500K, 2500K and 3000K. * p<0.05.

The distribution of PVT reaction times (number of observations from 100 to 500 ms of RT) revealed that light exposure at 6500K led to a shift towards the faster range of RT in the distribution curves in comparison to light at 2500K and 3000K (Fig. 5 first panel). For the PVT performance, main factor “light condition” elicited significance for median RT (F_2,12_ = 8.2, p<0.001), 10% fastest RT (F_2,12_ = 6.2, p<0.001) and 10% slowest RT (F_2,12_ = 7.4, p<0.05). Similarly, main factor “session” yielded significance for the median RT (F_2,21_ = 19.1, p<0.001), 10% fastest RT (F_2,21_ = 11.8, p<0.001) and for the 10% slowest RT (F_2,21_ = 13.7, p<0.001). No main effects for “light condition” and “session” were observed for the interpercentile range and for lapses (RT>500 ms). The analysis of variance for repeated measures (r-ANOVA) with the interaction of factors ‘light condition’ and ‘session’ yielded no significant differences in the slowest RT, interpercentile range and for lapses. On the other hand, significant differences were elicited for the median RT (F_4,11_ = 9.3, p<0.05; Fig. 5 second panel) and 10% fastest RT (F_4,11_ = 10.7, p<0.05; Fig.5 third panel), such that light at 6500K induced significantly faster reaction times in comparison to light exposure at 2500K and 3000K.

The GO/NOGO performance indicated that main factors “light condition” (F_2,14_ = 3.1, p = 0.05), “session” (F_2,21_ = 9.7, p<0.001). Furthermore, the interaction of these factors (2-way r-ANOVA F_4,13_ = 2.6, p = 0.04; Fig.5 bottom panel) yielded significance for RT, such that light at 6500K led to significantly faster reaction times in comparison to light exposure at 2500K and 3000K.

### Melatonin suppression

**Figure 6:**
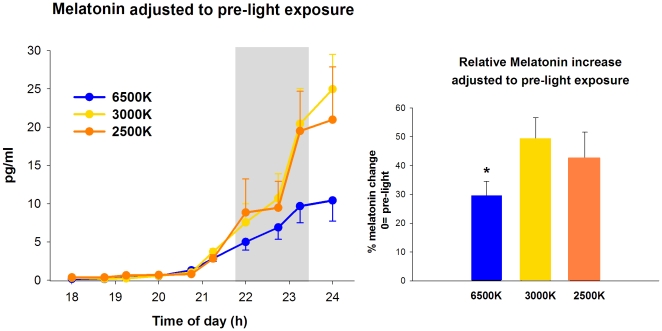
Salivary melatonin during pre-light, 2-h light (grey bar) at 6500K, 2500K and 3000K, and post-light. * p<0.05.

Light exposure caused a wavelength-dependent suppression of salivary melatonin, whereby light at 6500K resulted in attenuated melatonin secretion (adjusted to each individuals melatonin pre-light values) in comparison to the other light conditions (main factor “light condition”, F_2,23_ = 4.1, p = 0.04). These differences occurred mainly after 90 minutes of light exposure, and persisted during post-light exposure (Fig. 6). Comparison of salivary melatonin levels across different light conditions indicated that light at 6500K, 3000K and 2500K resulted, respectively, in an increase of 29.5±5%, 49±7.6% and 42±8.6% in comparison to pre-light exposure (1-way ANOVA, F_2,17_ = 2.1, p = 0.03).

**Figure 7:**
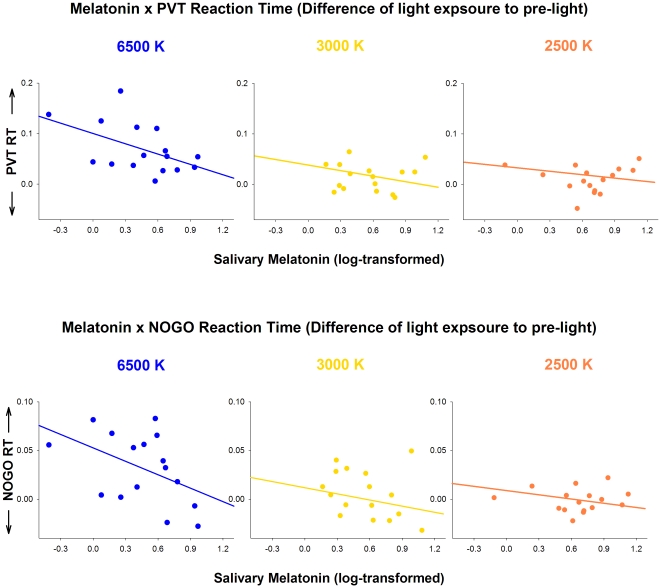
Correlations between salivary melatonin and PVT RT and GO/NOGO RT for light at 6500K, 2500K and 3000K.

Considering that light at 6500K led to an attenuated salivary melatonin expression, and faster performances in the PVT and GO/NOGO tasks, we performed correlations between these variables in order to verify if lower melatonin levels could underlie differences in these cognitive tasks. Salivary melatonin correlated negatively and significantly with PVT reaction times for light exposure at 6500K [R = −0.51; p = 0.04] and negatively and with a tendency for light exposure at 3000K and 2500K [respectively, *R* = −0.29; p = 0.09, and *R* = −0.23; p = 0.06]. Furthermore, salivary melatonin correlated negatively and significantly with the GO/NOGO reaction times for light exposure at 6500K [*R* = −0.46; p = 0.04], while although the correlation was negative, it did not reach statistical significance for light exposure at 3000K, and was a tendency for light at 2500K [respectively, *R* = −0.27; p = 0.16; *R* = −0.22; p = 0.07]. As depicted in Fig. 7 (upper and bottom panels), lower levels of salivary melatonin led to faster reaction times in both PVT and GO/NOGO performance during light at 6500K.

## Discussion

Here we demonstrate that the alerting response to polychromatic light in the evening is wavelength-dependent, such that light at 6500K is more effective than light at 2500K and 3000K in reducing subjective sleepiness and enhancing cognitive performance, specifically associated with tasks of sustained attention.

In our study, light exposure caused a wavelength-dependent suppression of salivary melatonin, such that light at 6500K resulted in a significant attenuated melatonin secretion, particularly after 90 minutes of light exposure, and which persisted during post-light exposure. This stands in agreement with recent findings suggesting that the human circadian pacemaker is highly sensitive to short wavelength light [6], [7], as indexed by action spectra for human melatonin suppression and assessment of human circadian phase resetting [30], [31]. The differential spectral sensitivity of non-image forming responses to visual responses [6], [7] has challenged the classical involvement of rod and cone photopigments in responses to light. Since Berson and co-workers [1] detected intrinsic photosensitive retinal ganglion cell (ipRGC) in the mammalian retina, it began to emerge that the eye plays a dual role in detecting light for a range of behavioral and physiological responses apart from the classical visual responses. Melanopsin-containing ipRGCs have a specialized non-visual retino-hypothalamic tract which provides direct neuronal connections to the suprachiasmatic nucleus (SCN), as well as direct and indirect (via SCN) projections to brain areas implicated in the regulation of arousal [32]. Furthermore, the SCN has connections to the pineal gland, which is responsible for the regulation of melatonin, as well as with many areas that share input from the visual photoreceptor system, such as the lateral geniculate nucleus, pretectum, and superior colliculus [33]. However, very recent findings suggest that cone photoreceptors also contribute substantially to non-visual responses at the beginning of a light exposure and at low irradiances, whereas melanopsin may be the primary circadian photopigment in response to long-duration light exposure and at high irradiances [8].

Our results on nocturnal melatonin decrease after light at 6500K thus go in line with the now established non-visual effects of light on the circadian system. However, while previous studies focused on monochromatic light exposure, we could demonstrate that even with light at 6500K from commercially available compact fluorescent lamps melatonin is suppressed by nearly 40% in comparison to traditional light lamps (3000K). Many studies have shown that exposure to white polychromatic light during night-time increases alertness [34], [35], [36], [37]. The dose-response relationship between light alerting effects and its irradiance is such that half of the maximum alerting response to bright light at 9100 lux can be obtained with room light of approximately 100 lux [36]. In contrast, our study revealed that light at 6500K from compact fluorescent lamps at low intensity (approximately 40 lux) was most effective after approximately 90 minutes of exposure. Although this luminance level is rather low to elicit such alerting effects, it is likely that the higher amount of blue component in the action spectra of light at 6500K may suffice to elicit these alerting effects. This finding adds up to the high specificity for light in the short wavelength range, and shows that the non-image forming visual system seems to crucially depend on light exposure at particular wavelengths.

Interestingly, we could demonstrate that exposure to blue-enriched light was most effective in enhancing cognitive performance for tasks of sustained attention, while no apparent benefit seemed to occur for higher executive cognitive tasks, such as the PVSAT. Light exposure impacts on cognitive performance through its synchronizing/phase-shifting effects on the circadian clock or acutely via its alerting effects, to the extent that performance in tasks such as digit recall, serial addition-subtraction and simple reaction time tasks can immediately improve after nocturnal light onset [35], [38], [39], [40]. Recent fMRI data supports light-induced modulations of cortical activity during auditory cognitive tasks for alertness-related subcortical structures, such as the brainstem [15], the hypothalamus, in a location encompassing the SCN [13], and dorsal and posterior parts of thalamus [12], [14], and amygdala [15]. These responses point towards a wide-range of subcortical and cortical networks activated by non-visual effects of light during specific cognitive tasks. While cognitive tasks associated with sustained attention can improve under short-wavelength light exposure, there remains some controversy with respect to higher executive functioning. fMRI assessed brain responses undergo a wavelength-dependency for higher executive task (2-back task), such that blue light (480 nm) enhances modulations in the brainstem (in a LC-compatible location), in the thalamus and insula, in relation to green (550 nm) and violet exposures (430 nm). However, while subcortical regions are activated faster and show short-lasting responses to light, cortical activity requires stronger and longer stimulations, as indicated in a study [12], in which 20 minutes of bright white light induced both thalamic and cortical modulations that steadily declined after light exposure albeit its rather lasting effects.

One critical finding in our study is that lower levels of salivary melatonin were strongly correlated with faster reaction times in tasks of sustained attention (PVT and GO/NOGO), particularly in the light at 6500 K condition. Interestingly, light at 6500K shifted the PVT distribution curve towards the faster range of RT in comparison to light at 2500K and 3000K. Thus, rather the optimal (i.e. 10% fastest RT) than the lapse domain of the PVT, which responds earliest to elevated homeostatic sleep pressure [41] was affected by light at 6500K. Similar effects in the optimal domain of the PVT (i.e. quicker 10% fastest RT) were reported after caffeine intake (200 mg), particularly in caffeine sensitive volunteers [42]. Thus, from a sustained attention perspective, blue enriched light has similar effects as caffeine such that both treatmens affect the optimal performance domain. In well-rested conditions, this optimal PVT domain has been associated with greater BOLD responses in the fronto-parietal sustained attention network [42]. This, in turn, allows for the allocation of attentional resources, by reflecting a top-down modulation of attentional for sustaining focus on a task [42]. Light irradiance signal reaches the SCN in the hypothalamus, and then the LC in the brainstem which triggers thalamic activity and a widespread modulation over cortical activity [43]. Given the significant inverse correlation of PVT RT with melatonin levels during light exposure at 6500K, one might speculate that enhanced activation in the sustained attentional network may be modulated by the non-visual system via the photopigment melanopsin.

When considering the implications of blue-enriched light on non-clinical settings, it is also important to take into account how this light setting can impact on subjective well-being. In our study, exposure to blue-enriched light improved subjective well-being, which has also been described for blue-enriched daytime light exposure [44]. Taken together, our results imply that alertness, subjective well-being and cognitive performance can be improved by enriching the spectral composition of light sources with short wavelengths.

Light-producing devices such as compact fluorescent lamps emitting more short-wavelength energy are effective at stimulating subjective and objective correlates of alertness and performance in the evening, which is correlated with the degree of concomitant suppression of endogenous melatonin levels. Since some of the effects persisted after light exposure, it is fair to suggest that conventional visual photoreception was not the major mediator of these responses. Furthermore, these light settings may provide an effective rationale for enhancing alertness and performance, as well as in the treatment for circadian rhythm sleep disorders, such as shift work disorder.
